# Pachydermoperiostosis: Classic Presentation of a Rare Disease

**DOI:** 10.31138/mjr.31.2.214

**Published:** 2020-06-30

**Authors:** Rajat Kharbanda, TG Sundaram, Latika Gupta

**Affiliations:** Department of Clinical Immunology & Rheumatology, Sanjay Gandhi Postgraduate Institute of Medical Sciences, Lucknow, India

**Keywords:** Clubbing, pachydermoperiostosis, primary hypertrophic osteoarthropathy, hypertrophic osteoarthropathy

A thirty-four-year-old gentleman reported with non-inflammatory knee arthralgias of 30 years duration. He presented with limitation of movement in both knees for the past one year. He had experienced mechanical pain in the ankles and feet on occasion. He was a non-smoker with an unremarkable history.

**Figure 1(A).**
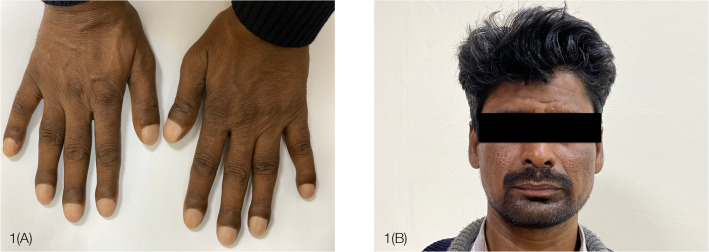
Image depicting clubbing of all digits. **Figure 1(B).** Image of prominent folds on the patient’s forehead.

On examination, the prominent folds of skin were noted on the patient’s forehead (*Figure 1A*), apart from an oily facial skin and grade four clubbing of all fingers (*Figure 1B*) and toes. Terminal knee extension was restricted. The right ankle was swollen and tender with painful limitation of terminal movement, although movement was preserved on the left side, which was swollen but non-tender. Considering the onset of clubbing in childhood, with a family history of similar manifestations in the absence of lung or cardiac disease, a diagnosis of primary hypertrophic osteoarthropathy (pachydermoperiostosis) was deemed likely.

**Figure 2(A).**
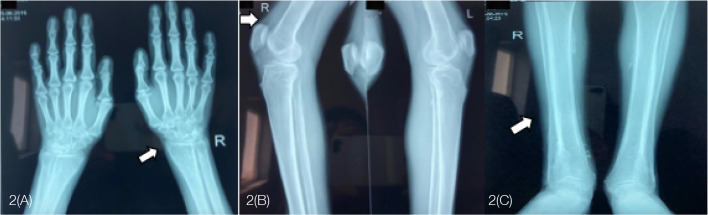
Radiograph of hand demonstrating widening of distal end of radius and ulna (white arrow). **Figure 2(B).** Radiograph of knee depicting irregular cortical thickening of bilateral femur (white arrow). **Figure 2(C).** Radiograph of legs showing sub-periosteal bone formation (white arrow).

On further review, he reported noting disproportionately larger hands and feet as compared with peers, a perception shared by his sister as well. Besides, his sister has a similar affliction of the fingers. Laboratory investigations and chest radiograph were unremarkable. The radiograph showed irregular cortical thickening of the bilateral femur, the sub-periosteal bone formation of proximal tibia and fibula, with irregular-subperiosteal bone formation and cortical thickening of the distal tibia, fibula (*Figures 2B,C*). Radiographs of bilateral hands showed enlargement of distal ulna and radius, metacarpals, phalanges (*Figure 2A*). He was prescribed Nonsteroidal anti-inflammatory drugs (NSAIDs) with a plan to consider bisphosphonate in the event of inadequate response. On follow up visits, the patient had relief in his articular symptoms.

Pachydermoperiostosis, also known as Touraine-Solente-Golé syndrome, is an autosomal-dominant disorder with variable penetrance. In its complete form, it is characterized by pachyderma (thickening of the facial skin), skeletal changes (periostosis), excessive sweating (hyperhidrosis), and acropachy (digital clubbing).^1,2^ Pachydermia is the most frequent skin symptom. Digital clubbing occurs in 89% of cases, and the classic radiologic findings in 80–97% of patients.^3^ Borochowitz and Rimoin^1^ proposed the presence of at least two of the four criteria - a history of familial transmission; pachydermia; digital clubbing, and skeletal manifestations such as pain or signs of radiographic periostitis for diagnosis. This patient fulfilled all four criteria.

The differential diagnoses include lung cancer, acromegaly, psoriatic arthritis, carcinomatous polyarthritis, thyroid acropachy, fluorosis and hypervitaminosis A.^4^ With classic findings, and no history or clinical findings explaining any of the above diagnoses, the diagnosis was never in doubt. Since prostaglandin E2 plays a central role in the pathogenesis of this disease, NSAIDs are the first line of treatment.^5^ Pamidronate is reportedly effective in NSAID-resistant cases.^6^
